# The epidemiology of swine influenza

**DOI:** 10.1186/s44149-021-00024-6

**Published:** 2021-09-28

**Authors:** Yin Li, Ian Robertson

**Affiliations:** 1 School of Veterinary Medicine, Murdoch University, Perth, WA Australia; 2 Commonwealth Scientific and Industrial Research Organisation, St. Lucia, QLD Australia; 3 College of Veterinary Medicine, Huazhong Agricultural University, Wuhan, 430070 China; 4 Hubei International Scientific and Technological Cooperation Base of Veterinary Epidemiology, Huazhong Agricultural University, Wuhan, 430070 China

**Keywords:** Swine influenza, Influenza, Risk factors, Epidemiology, Zoonotic transmission

## Abstract

Globally swine influenza is one of the most important diseases of the pig industry, with various subtypes of swine influenza virus co-circulating in the field. Swine influenza can not only cause large economic losses for the pig industry but can also lead to epidemics or pandemics in the human population. We provide an overview of the pathogenic characteristics of the disease, diagnosis, risk factors for the occurrence on pig farms, impact on pigs and humans and methods to control it. This review is designed to promote understanding of the epidemiology of swine influenza which will benefit the control of the disease in both pigs and humans.

## Introduction

Swine influenza (SI) is a respiratory disease of pigs caused by influenza A viruses. The typical clinical signs associated with the disease include coughing, laboured breathing, nasal discharge, sneezing and pyrexia (Kothalawala et al. 2006) and lesions of pneumonia may be observed in infected pigs at slaughter (Karasin et al. 2000; Vincent et al. 2008; Rose et al. 2013). Reproductive problems, including abortion and stillbirths, have also been reported in sows infected with swine influenza virus (SIV) (Wesley 2004). Subclinical infection is common, especially in herds with antibody against homologous SIV strains (Choi et al. 2004; Rose et al. 2013; Hemmink et al. 2016).

Influenza A viruses belong to the Orthomyxoviridae family, which are enveloped viruses containing eight single strand RNA segments. Subtypes of influenza A viruses are determined by antigenic and genetic properties of two major viral envelope proteins: hemagglutinin (HA) and neuraminidase (NA) (Hause et al. 2014). Currently, there are 18 HAs (H1-H18) and 11 NAs (N1-N11) recognised, with H17–18 and N10–11 types having only recently been isolated from bats (Mehle 2014). Influenza A viruses are the most clinically important influenza viruses as they can cause serious disease in a wide range of species, including humans, pigs, birds, horses, cattle, whales, seals, tigers, dogs, cats and ferrets (Mehle 2014). There are three other genera of influenza viruses: Influenza B, C and D. Influenza B viruses have mainly been isolated from humans and seals (Osterhaus et al. 2000). Influenza C viruses are primarily found in humans, pigs and dogs, and influenza D has recently (2011) been detected in pigs and cattle (Hause et al. 2014; Luo et al. 2017).

Most avian influenza viruses’ HA bind specifically with α-2, 3-galactose sialic acid, which is abundant in epithelial cells of avian trachea, while most human influenza viruses prefer α-2, 6-galactose sialic acid linkage, which is abundant in epithelial cells of human trachea. However epithelial cells of pig trachea have both α-2, 3- and α-2, 6-galactose sialic acid linkages, and so pigs can be susceptible to both avian and human influenza viruses (Ito et al. 1998). Pigs are believed to act as a “mixing vessel” for swine, avian and human influenza viruses allowing the production of reassortant influenza viruses.

Swine influenza has huge economic impact on pig industry as it is prevalent and can cause serious loss when associated with other disease (Lin et al. 2016; Rech et al. 2018). In addition, the disease is also a zoonosis. Despite the potential impact of SIV on the pig industry and public health, SI is often neglected by the pig industry workers. The main reason for this is that many of infections in pigs are subclinical or mild and hence easily overlooked (Detmer et al. 2013). Furthermore, although the morbidity of SIV infection in a herd can be as high as 100%, mortality is usually extremely low (Er et al. 2014). Although many studies has been carried out on SI at molecular level, there are still knowledge gaps in this disease (Li et al. 2020b; European Union 2021). For example, what have facilitate the spill-over infection to human? What are the environmental and anthropological risk factors for SI infection in pigs and human? More research especially epidemiological studies are needed to answer these questions.

## Swine influenza viruses

### Characteristics of swine influenza viruses

As a member of the influenza A virus group, SIV is an enveloped virus with eight segments of RNA (HA, NA, PA, PB1, PB2, NP, M and NS) (Reeth et al. 2012). The infectivity of the virus is mainly determined by two proteins, HA and NA. HA can bind receptors in host and facilitate virus invasion into host cell while NA is typically responsible for cleaving sialic acid so the reproduced virus particles can release (David et al. 2020). However, NA may also contribute to binding host cells (Benton et al. 2017).

Influenza viruses are sensitive to environmental conditions. Chemical disinfectants such as 0.1 mol/L NaOH, 70% ethanol, 70% 1-propanol and ethylene oxide can effectively inactivate them (Jeong et al. 2010). One study reported that even a powdered laundry detergent with peroxygen (bleach) was sufficient to kill the virus (Lombardi et al. 2008). However, the inactivating efficiency of many disinfectants is reduced at low temperatures and in environments contaminated with organic material, and consequently, caution is needed when disinfecting SIV-infected premises during winter and organic matter should be removed prior to disinfection (Haas et al. 1995; Botner and Belsham 2012). The infectivity of SIV can be retained for more than six weeks in slurry at 5 °C, whilst at 20 °C it can remain viable for up to 14 days (Botner and Belsham 2012).

### Subtypes and genetic recombination among strains

Many subtypes of influenza A virus have been isolated from pigs and continue to circulate and diversify (Lewis et al. 2016). Among these subtypes, the most common SIVs circulating in pig populations are subtypes H1N1, H3N2 and H1N2. The dominant strains in the USA are 1A classic H1N1, triple reassortant H3N2, 1C avian-like swine H1N1 and pandemic A/H1N1 2009 (1A H1N1pdm09) virus (Bowman et al. 2014b). In contrast, in Europe the dominating strains are 1C avian-like swine H1N1, 1B human-like reassortant swine H1N2, human-like reassortant swine H3N2, and 1A H1N1pdm09 virus (Simon et al. 2014; Watson et al. 2015b; Anderson et al. 2016); however in China all of the lineages from both the USA and Europe have been shown to be circulating in pig population (Chen et al. 2014a; Xie et al. 2014; Yang et al. 2016).

Co-circulation of different SIV strains is commonly seen in piggeries. Active surveillance undertaken in the USA reported simultaneous infection with influenza H3N2 and H1N1pdm09 virus in 8 different age categories of pigs from four to over 24 weeks of age (Corzo et al. 2013a). Another study in Italy reported infection with multiple reassortant genotypes of H1N2 in one local commercial breeding farm (Beato et al. 2016). In south China, 24% pigs tested from seven of nine counties surveyed were positive for both H1 and H3 (Song et al. 2010).

The coexistence of different SIV strains within individual pigs facilitates gene reassortment and emergence of new strains. A cohort study in three selected farrow-to-finish pig farms in France found that H1N1 and H1N2 viruses could simultaneously existed in the same farm, batch or even individual pigs, and reassortants between viruses from these lineages could be isolated from infected herds (Rose et al. 2013). After the pandemic of “swine flu” in 2009, gene reassortment between H1N1pdm09 viruses and local endemic swine viruses were identified in many countries, including the USA, Brazil, Germany, Italy, UK, Vietnam, Thailand, Japan, Korea and China (Abe et al. 2015; Kong et al. 2015). Whole-genome phylogenetic analysis of 368 influenza A viruses circulating in the USA has demonstrated the presence of 44 different genotypes of H3N2 from 2009 to 2016, with the majority of these genotypes containing at least one gene segment from H1N1pdm09 (Rajao et al. 2017).

Swine influenza virus reassortants can become endemic in pig farms and potentially transmit to humans, resulting in pandemic circulation. The best-known example is 2009 H1N1 pandemic influenza A virus involving a reassortment of three different influenza strains circulating in pigs, birds and humans (van der Meer et al. 2010). Shortly after the outbreak of H1N1 in the USA, H1N1pdm09 virus was found in pig and human population all over the world. A survey undertaken in north Vietnam in 2009 reported a maximum seroprevalence of H1N1pdm09 of 55.6% (95% CI: 38.1–72.1) in pigs sampled at a slaughterhouse, with a farm-level seroprevalence of 29% (95% CI, 23.2–35.7) (Trevennec et al. 2012). In China, H1N1pdm09 virus was also first isolated in pigs in 2009, and reassortants with internal genes from the pandemic 2009/H1N1 viruses were found in pigs in the following years (Chen et al. 2013; Chen et al. 2014b; Qiao et al. 2014). H3N2 variants containing genes from H1N1pdm09 influenza virus were subsequently isolated from at least seven countries between 2009 and 2013, and it seems to be the most commonly emerging SIV genotype (Kong et al. 2015). It was suspected that H1N1pdm09-origin internal gene segments had fitness advantage over the segments of other SIV’s in terms of contributing genes for new reassortants (Kong et al. 2015).

## Distribution of swine influenza

Swine influenza was first observed in 1918 in the USA, Hungary and China (Brown 2000), and today it is one of the most ubiquitous diseases circulating in the global pig population. Corzo et al. (2013a) reported an individual prevalence level of 4.6% in the USA pig population, and a 90.6% herd prevalence in the participating farms with a period of 12–24 months using a real-time reverse transcription polymerase chain reaction (real-time RT-PCR). A cross-sectional study in northern Mexico reported that more than 50% samples tested from commercial farms with between 300 and 2500 sows were seropositive to either H1 or H3 subtype SIV (Lopez-Robles et al. 2014). However, the seroprevalence may have been overestimated because these authors primarily sampled pigs less than ten weeks of age and maternally derived antibody (MDA) can last up to ten weeks in pigs and potentially have resulted in false positive results (Cador et al. 2016). This assumption was also supported by the finding of decreasing antibody titres with increasing age of the sampled pigs. The authors also reported that 16.7% (25/150) sampled pigs were positive for type A influenza with a RT-PCR test (Lopez-Robles et al. 2014).

Swine influenza is also widespread in Europe. In study in Belgium, France, Italy and Spain, 80 farrow-to-finish farms were monitored from 2006 to 2008. Ninety percent farms were classified as positive for SIV, with an individual level seroprevalence of 62%. Forty-nine percent of farms were infected with one subtype, 38% with two subtypes and 3.9% with three subtypes of SIVs (Kyriakis et al. 2013). However in this study, the sampling was also biased, resulting in potential overestimation of the prevalence as farms sampled were selected from areas with a high density of pigs or contained pigs that had a history of respiratory problems. An analysis of historical surveillance data in Norway showed that the national herd seroprevalence of influenza A(H1N1)pdm09 virus was around 43%, and the individual pig prevalence of pandemic H1N1 in infected farms was more than 60% (Er et al. 2016a). Another study in Spain in 2009, that involved sampling pigs from 98 randomly selected pig farms, reported a farm-level seroprevalence of nearly 100% with an animal level seroprevalence of 62.3% (Simon-Grife et al. 2011). In England, a 52% herd prevalence was reported by Mastin et al. (2011), with the highest individual prevalence of 33% being reported in sows.

Swine influenza is enzootic in the Chinese pig population, with many subtypes contemporarily circulating on farms. Serological evidence indicateed the presence of H1, H3, H4, H5, H7 and H9 influenza viruses in pig populations in the country (Ninomiya et al. 2002; Liu et al. 2011; Yu et al. 2011; Ding et al. 2021). SI is also prevalent in pig populations in other countries in South East Asia (Lewis et al. 2016). H1N1, H1N2 and H3N2 subtypes are cocirculating in pig population in Vietnam, and spill-over infection of human origin influenza viruses to pigs were detected. Notably, some SIV reassortants had infected humans (Takemae et al. 2017; Baudon et al. 2018; Takemae et al. 2018; Baudon et al. 2020). Similarly, the three subtypes are also prevalent in the pig population of Thailand and A(H1N1)pdm09 and its reassortants are found as the predominate strains in the field (Nonthabenjawan et al. 2015; Mine et al. 2019; Nasamran et al. 2020).

## Diagnosis of swine influenza

### Serological methods

Serological tests for SIV mainly target host antibodies against the virus. The most commonly used serological tests are hemagglutination inhibition (HI) test and enzyme-linked immunosorbent assay (ELISA). Many commercial ELISA kits have been developed to detect antibody to influenza A nucleoprotein (NP) because it is highly conserved in influenza A viruses (Goodell et al. 2016). Several studies have shown that an NP blocking ELISA kit for testing antibodies in birds can also be used to detect NP antibodies in pigs (Nava et al. 2013; Goodell et al. 2016). In general, HI tests are simpler to operate, cheaper and quicker than ELISAs; however choice of antigen is complex because of the diversity of SIV. In addition, the sensitivity of HI test can be low if used solely for SIV surveillance when heterologous viruses are present (Goodell et al. 2016), although they do offer the advantage that they can be used for subtyping viruses (Van Reeth et al. 2006). It worth noting that red blood cells from different species such as chicken, turkey and guinea pig can be used for HI test, and the erythrocyte species can impact titers of HI test against SIVs (Wiriyarat et al. 2010; Ovsyannikova et al. 2014; Trombetta et al. 2018).

Serological surveys/tests often take advantage of existing collections of serum samples, as collecting blood samples involves significant cost, time and labour inputs. To overcome this, a new method targeting the antibody in the oral fluid of swine, using a NP-blocking ELISA, has been developed (Panyasing et al. 2014). This test would only give a population-level assessment of a herd. With experimentally infected pigs, NP antibodies in the oral fluid were detected 7 to 42 days post-infection in all challenged groups. The oral fluid versus serum sample-to-negative (S/N) ratios from pigs in the same pen showed a correlation of 0.796, indicating good agreement between results for testing oral fluid samples and serum samples (Panyasing et al. 2014). In contrast, another study that used field-collected oral samples found that NP blocking ELISA had a much lower sensitivity in 10–14-week-old pigs compared with matched serum samples (19% for oral fluid and 93% for serum, *P* < 0.01) (Gerber et al. 2017).

There are several advantages with using serological tests: firstly, they are often inexpensive; secondly, they are easier to perform compared with polymerase chain reaction (PCR) tests or virus isolation (VI); and thirdly, serological tests are more sensitive in detecting exposure of pigs to influenza A virus than PCR tests or virus isolation because the antibodies can last for at least 1.5 months post-infection, and consequently, serological tests are less sensitive to the sampling time (Goodell et al. 2016). However serological tests have limitations which include: they only provide information on historical exposure to SIV and do not provide viral genetic information or live viruses which are vital for evaluating the potential pandemic threat of strains; cross-reactions can occur between different lineages within one subtype, or even among different subtypes; and maternally derived antibodies may interfere with the accuracy of the test (Allerson et al. 2013a; Detmer et al. 2013).

### Molecular methods

Molecular tests of nasal swabs, nasal wipes or oral fluids are mainly used in surveillance to detect the presence of SIV RNA and to produce amplicons for further sequencing. Conventional molecular assays for influenza A viruses target a conserved amplicon of M-gene. For subtyping, specific primers need to be designed to detect different gene segments, mainly *HA* and *NA* (Henritzi et al. 2020). Universal primers can also be used to amplify cDNA, which is then sequenced (Inoue et al. 2010).

Real-time RT-PCR assays for SIVs detection were first developed in 2004 (Richt et al. 2004). Compared with conventional RT-PCR, it can be performed in a shorter time (within a few hours) and can differentiate SIV subtypes. It can also be less expensive than VI and conventional RT-PCR assays. Most importantly, real-time RT-PCR doesn’t require post–PCR sample handling, thus reducing the potential for cross-contamination (Richt et al. 2004).

For public health purposes, detection of coinfection with different virus strains in a pig herd would be very valuable in SIV surveillance. Multiplex RT-qPCR assays can differentiate H1, H3, N1 and N2 SIV subtypes. These multiplex RT-qPCR assays can also identify different lineages within H1 subtype, such as 1C “av” (European avian-derived), 1B “hu” (European human-derived) and 1A “pdm” (H1N1pdm09). Henritzi et al. (2016) reported that multiplex RT-qPCR assays that they developed could detect double infections with different lineages in one clinical sample. However, efficiency of RT-qPCR relies heavily upon the specific primers having no mismatches with target amplicon sequence, with outdated primers resulting in low test sensitivity (Yang et al. 2014). Since the primers used by Henritzi et al. (2016) were designed specifically for SIV strains circulating in Europe, whether these RT-qPCR assays could be used for SIV surveillance in other regions/continents requires further study.

### Virus isolation

Isolation of SIV is undertaken routinely in embryonated chicken eggs (ECEs) and various cell lines, including Madin-Darby canine kidney (MDCK) and CACO-2 cell line (Chiapponi et al. 2010). It has been reported that sensitivity of SIV isolation with different methods is dependent upon the virus strains present. A study using strain A/Swine/Indiana/1726/88 (H1N1) showed that ECE was more sensitive than MDCK cell line (Clavijo et al. 2002). In contrast, in another study with clinical samples, use of MDCK cell line resulted in recovery of more isolates of H1N2 and H3N2 than with ECE (Bowman et al. 2013), whilst CACO-2 line was shown to be more sensitive (*p* < 0.01) for the isolation of H1N1 and H1N2 subtypes in Italy compared to both MDCK cells and ECEs (Chiapponi et al. 2010). However for H3N2 virus, isolation in ECE has been demonstrated to be better than in cultured cells (p < 0.01) (Chiapponi et al. 2010).

VI is often difficult, expensive and time consuming, but it is necessary when the live virus is required for further research, such as evaluating the pathogenicity of new SIVs and screening for vaccine candidate strains (Detmer et al. 2013).

In conclusion, serological tests can be used in SI surveillance to tell exposure history of pigs to influenza virus; PCR tests are more suitable for detecting the presence of SIVs and the acquired gene material can be used for molecular studies; VI is often necessary if live viruses be needed for further research such as vivo test and vaccine development.

## Epidemiology

Swine influenza is endemic in many countries in North and South America, Europe, Asia and Africa (Almeida et al. 2017). Infection in pig farms can be seen throughout the year, although an increased number of cases are often seen in spring and winter (Beaudoin et al. 2012; Kyriakis et al. 2013). It is believed that commercial pig farms have a higher risk of infection compared to backyard farms, especially for infection with new SIV reassortants (Gonzalez-Reiche et al. 2017). Farrow-to-finish pig farms are more susceptible to SIV infection than fattener enterprises because they continuously produce naïve piglets (Loeffen et al. 2003; Kyriakis et al. 2013). In an infected herd, sows have the highest risk of being seropositive, most likely linked to their older age resulting in greater opportunity for exposure to the virus, while the greatest chance of isolating live viruses is from piglets (Mastin et al. 2011; Takemae et al. 2011; Ozawa et al. 2015; Er et al. 2016a).

Virus transmission between pigs is mainly through direct pig-to-pig contact. Aerosol transmission is one of the common ways of indirect transmission of SIV (Brown 2000; Corzo et al. 2013b; Hemmink et al. 2016). A pig farm can become infected through the introduction of carrier pigs or entry of the virus on infectedvisitors, or on vehicles or other fomites (Simon-Grife et al. 2011; Allerson et al. 2013b; Er et al. 2016a), highlighting the importance of strict farm biosecurity.

In infected pigs, SIV is excreted in oral and nasal secretions, with no virus shed in the faeces (Choi et al. 2004; Botner and Belsham 2012). Pigs can start to shed virus within 2 days of infection. Although the duration of shedding is usually 8 to 10 days, shedding for more than 30 days has been reported (Choi et al. 2004; Botner and Belsham 2012). The reason for a long shedding period has been postulated to be linked to the suppression of immunity in infected pigs (Choi et al. 2004).

Between individual pigs within a herd, the transmission of SIV can be rapid. Rose et al. (2013) reported that in farrow-to-finish pig farms with recurrent influenza outbreaks and no prior immunity, the basic reproduction value (R0) was high, between 2.5 and 6.9.

## Interspecies transmission

HA subtypes circulating between birds, pigs and humans include H1-H16, with different subtypes predominantly circulating in individual species. Wild waterfowl are the natural reservoir of H1-H16, while domestic chickens are mainly infected by H5, H7 and H9 subtypes. For humans and pigs, the most common circulating subtypes are H1-H3 and H1 and H3, respectively (Short et al. 2015). Pigs can contract influenza A viruses from other species, especially from infected humans and birds (Karasin et al. 2000; Grontvedt et al. 2013; Nelson and Vincent 2015).

Avian influenza viruses have been isolated from pigs in many countries and regions. In Canada, H4N6 was isolated from pigs with pneumonia on a commercial swine farm and similarly an avian-origin H4N6 was isolated from pigs displaying clinical respiratory signs in the USA in 2015 (Karasin et al. 2000; Abente et al. 2017). A study in Nigeria reported that 22 of 129 samples collected from apparently healthy pigs were positive to H5N1. At the same time, sampling there showed an incursion of highly pathogenic avian influenza (HPAI) H5N1 in local poultry (Meseko et al. 2018). In addition, both avian H9N2 and H5N1 viruses were detected in pigs in Egypt in 2014 and 2015 (Gomaa et al. 2018). In China, 28 isolates of H9N2 were detected in pigs from 1998 to 2007 (Yu et al. 2011). The isolates of H9N2 AIVs recently detected circulating in poultry farms in south China have shown increased ability to replicate in pigs than did earlier isolates (Sun et al. 2019), highlighting the greater risk of new viral reassortants appearing in this location.

In pigs, infection with human origin influenza A virus appears to be more common than that from avian-origin influenza A virus (Nelson and Vincent 2015). A study in the Czech Republic reported the presence of antibodies against human influenza virus isolated during the 1995 epidemic in local pig population. It is possible that the human virus was introduced to pig herds by infected animal attendants, in whom antibodies against this virus were also found (Pospisil et al. 2001). In China, former prevailing human H1N1 strains have also been found to be circulating within pig population (Yu et al. 2009) and phylogenetic analysis indicated that infection arose through transmission from humans to pigs. It was interesting that in that study, 4 out of 5 virus isolates were from Guangdong province. This may be either because Guangdong actually had more pig infections arising from human influenza than other provinces, or that Guangdong had contributed more of the 500 tested samples. Although the samples were sourced from 8 different provinces, unfortunately the actual sample size from each province was not given (Yu et al. 2007). Introductions of human seasonal influenza viruses into pigs from 1965 to 2013 has been summarized by Nelson et al. (2015a), and the authors concluded that more than 40 cases of human-origin H1N1 viruses in pigs had been reported in the 5 years after H1N1pdm09 was initially detected in humans. H3N2 viruses closely related to human viruses that circulated in 2010 have also been found in pigs from Central America in 2010 (Gonzalez-Reiche et al. 2017).

Many researchers believe that most subtypes of influenza A viruses from other species are capable of transiently infecting pigs. However, as the majority of these strains have not been repeatedly detected in the same pig farms or in samples collected from pigs processed at slaughterhouses, it is assumed that they are not able to establish in pig population (Pospisil et al. 2001; Vijaykrishna et al. 2011; Li et al. 2015).

## Risk factors for SIV infection on pig farms

### Husbandry factors

Some management and husbandry practices are associated with SIV infection in pig farms. For example, piggeries that failed to prevent the entry of wild birds (OR 2.50, 95% CI: 1.01–6.16) and keeping poultry on the farm (OR 3.24, 95% CI: 1.52–6.94) were shown to increase the risk of SI infection in pig farms in South China (Li et al. 2019). Larger farms have been reported to have an increased risk of infection than smaller herds (Mastin et al. 2011; Takemae et al. 2016; Gonzalez-Reiche et al. 2017). A high density of weaners has also been shown to increase the risk of infection in herds (OR: 2.9; 95% CI: 1.2–7.0), as has failure to adopt an all-in all-out practice in the fattening room (OR = 2.4, 95% CI: 1.0–5.8) (Fablet et al. 2013). Another study reported that herds with a high number (> 18) of finishers per water space had an increased risk of infection (OR 5.22; 95% CI: 1.57–17.43) compared to herds with lower numbers of pigs (≤ 18) per water space (Mastin et al. 2011). In addition, one study found that the presence of open partitions between pens increased the risk of infection (Simon-Grife et al. 2011), most likely associated with increased contact opportunities between pigs. Other factors which have also been linked with an increased risk of SIV infection in pig herds include increased replacement rates in pregnancy units, farm type (farrow-to-finish and breeder herds had a higher risk of SI infection than finisher farms), having a suckling period of less than 28 days (for prevalence in weaners) and a fully slatted floors in pens (Simon-Grife et al. 2011; Baudon et al. 2017). Low room/ambient temperature (< 25 °C) in farrowing room has also been reported to increase the risk of infection (Fablet et al. 2013). In the United Kingdom, intensively housed (indoors) pigs had a higher risk of SIV infection than farms adopting extensive or outdoor housing (Mastin et al. 2011). Similarly the use of straw yards in UK farms has been shown to reduce the risk of infection (Mastin et al. 2011; Fablet et al. 2013). In conclusion, the husbandry and management practices that would facilitate interactions between naïve pigs and those that would result in stress to pigs are potential risk factors for SI infection in pig farms.

### Biosecurity factors

Poor biosecurity nearly always leads to a higher risk of a range of diseases (Robertson 2020), including SI (Filippitzi et al. 2018). Some biosecurity factors have been reported to be associated with increasing SIV infection in pig farms. Firstly, frequent human-pig interaction increases the potential spillover of human influenza viruses from humans to pigs. One study in China found that although local pig industry workers had extensive close contacts with pigs, they had limited knowledge and awareness that SIV could infect humans (Li et al. 2020a). One study demonstrated that the presence of farm staff with influenza-like illness was significantly associated with the presence of SIV on pig farms in Norway (OR = 4.15, 95% CI 1.5–11.4, *p* = 0.005) (Grontvedt et al. 2013). A lower herd-level seroprevalence in Norwegian fattening herds was believed to be associated with fewer close human-pig interactions, in contrast to sow (breeding) herds which had the highest seroprevalence because sows frequently contacted with many different people (Er et al. 2016a). Secondly, uncontrolled access to the farm by vehicles or visitors can increase the chance of introducing diseases through contaminated vehicles, clothing, footwear and fomites. It was found to be a risk factor for H1N1 seropositivity (OR = 2.44, 95% CI: 1.01–5.87) in a study conducted in Spain (Simon-Grife et al. 2011). A third factor is disease management within the farms. Mastin et al. (2011) reported that the management of the sick pen was important; stating that the location of sick pens in a separate building to those housing healthy pigs may help reduce SIV infection, although this was not confirmed through a formal study. However, as with most infectious diseases, isolation of affected animals is a key management procedure to minimise transmission to other animals and contamination of environment (Cui and Chen 2017; Robertson 2020).

Most of the studies on risk factors for infection with SI have found agreement in risk and protective factors, although some studies did generate conflicting results. For example, Simon-Grife et al. (2011) reported that the presence of other species, such as cats, dogs, birds or cattle, on the farm increased the infection risk; in contrast Takemae et al. (2016) found that the presence of other animals on the farm was potentially protective. These conflicting results may be due to the different ecosystems and the different husbandry practices adopted in the surveyed herds, different populations under study or differences in the case definitions used in the individual studies.

### Environmental factors

Environmental factors for SIV infection have rarely been studied. However, the density of pig farms in an area appears to be a risk factor for SIV infection. Using machine learning modelling, one study in south China showed that pig density is one predictor variable for three influenza infection scenarios in pigs (infection with human strains, infection with avian strains, and coinfection with H9N2 avian strain and at least one swine strain) (Ding et al. 2021). Pasma (2008) analysed H3N2 SI outbreaks in Canada during the autumn of 2004 and found clustering of outbreaks in a region with a high pig density. It was hypothesized that the density of pig farms was a factor in clustering and spread of this outbreak, although the data didn’t show statistical significance for this factor. Couacy-Hymann et al. (2012) also thought low pig density in Côte d’Ivoire, Benin, and Togo might be the reason for low prevalence of avian and swine influenza in those three African countries. In addition, Ding et al. (2021) demonstrated that other environmental factors, including chicken density, duck density, human population density, annual cumulative precipitation and elevation, were predictor variables for infection of pigs with human/poultry influenza viruses.

Some studies on avian influenza have highlighted the role of environmental and meteorological factors in avian influenza outbreaks. Potential risk factors, such as monthly average rainfall in the preceding 3–7 months, being close to rivers, lakes or seacoasts, low ambient air temperature, and high relative humidity have been reported to be linked with avian influenza outbreaks (Fang et al. 2005; Si et al. 2013; Zhang et al. 2014; Ferenczi et al. 2016). Since pigs may also contract avian-source influenza viruses, these environmental and meteorological factors could also be potentially associated with outbreaks of SI and require further investigation. The study of Ding et al. (2021) offered evidence to support this hypothesis.

## Impact of swine influenza on the pig industry

### Morbidity and mortality

Swine influenza is a highly contagious disease with almost 100% exposed pigs becoming infected, although the mortality rate is usually very low. Even with infection in a naïve pig population, clinical signs may only be observed in a small proportion of pigs. Er et al. (2014) reporting that less than 7% pigs displayed clinical signs in an outbreak of a boar testing station in Norway.

However serious morbidity and mortality, associated with economic losses can occur when SIV simultaneously infects pigs with other swine diseases or when infection occurs in sows during the late stages of pregnancy (Fablet et al. 2012). A study reported that co-infection with *Mycoplasma hyopneumoniae* exacerbated the clinical effects of H1N1 infection (Deblanc et al. 2013). Wesley (2004) observed stillbirths in naturally infected gilts after challenge with live H3N2 SIV at 80 to 82 days of gestation. The average percentage of stillbirths was 22% per litter while the control gilts (also naturally infected but not challenged with live H3N2 SIV) had no stillbirths. Furthermore, abortions can also occur when sows are infected with new emerging strains of SIV (Gumbert et al. 2020).

### Productivity losses

The productivity losses caused by SIV infection include decreased feed conversion efficiency (FCE) and slower growth of pigs. Er et al. (2014) recorded an outbreak of H1N1pdm09 in a Norwegian boar station and analysed the infection on production performance in the resident pigs. Their study showed that seropositive and virus-positive pigs overall had reduced (*P* < 0.05) growth performance compared to seronegative pigs, even though the feed intake was not decreased. For seropositive pigs, the negative effect on growth performance was seen during growth from 81 to 100 kg (GF3), whereas FCE was reduced requiring an extra 0.029 kg of feed for every 1 kg of weight gain and the average daily growth (ADG, weight gain in kg/day) decreased an average of 0.015 kg/day. For virus-positive (with RT-PCR test) pigs, infection reduced ADG by 0.058 to 0.015 kg/day and also reduced FCE (an extra 0.058 to 0.125 kg of feed required for each kg of weight gain). Thus, infection resulted in an additional 2.3 kg and 5.9–8.0 kg feed for seropositive pigs and virologically positive pigs to reach 100 kg bodyweight, respectively. The virus-positive pigs also took an extra 1.6 to 2.4 days to reach 100 kg bodyweight. This delay in reaching market weight would also increase the cost of the disease.

Er et al. (2016b) estimated that a batch of 150 pigs infected with H1N1pdm09 in Norway would consume an extra 835 (fifth percentile) to 1350 kg (95th percentile) feed and take 194 (fifth percentile) to 334 (95th percentile) more pig days to reach expected body weights than an uninfected batch of 150 pigs. They also found that infection in the late stage of fattening could induce the greatest losses.

## Impacts on public health

### Swine-source influenza outbreaks and its prevalence in human

SIVs have a distinct impact on the potential for pandemic influenza in humans with 19 influenza A virus reassortants emerging in humans since 1918. Of these, three were predominantly zoonotic swine influenza A virus variants (Bui et al. 2017). Several swine-to-human spillover infections have been reported in China, as well as in other countries. One child infected with swine influenza H3N2 virus was reported in Hong Kong in 1999 (Gregory et al. 2001). Zu et al. (2013) reported a human case infected by European avian-like swine H1N1 influenza virus in Jiangsu province, with the same virus being isolated from the patient’s pigs in a smallholding belonging to the patient. Killian et al. (2013) investigated an outbreak of H1N1 at an Ohio county fair in the USA in 2007 and detected a triple-reassortant swine H1N1 influenza virus that had infected both people and pigs.

Human-adapted SIVs can result in pandemic circulation. The H1N1pdm09 affected 10–20% of humans globally and was a new strain incursion into swine and humans (Garten et al. 2009; Short et al. 2015). In Mexico in the period 2007–2008, 12.9 and 3.2% pig farm workers were seropositive to H3N2 and H1N1 SIV, respectively (Lopez-Robles et al. 2012). Ma et al. (2015) reported that in China, 17.3 and 7.0% workers in piggeries and workers in other occupations, respectively were also seropositive to swine H3N2 virus. However, cross-reactions between antibodies against human seasonal H3N2 and swine H3N2 can hamper interpretation of results. These studies did not rule out this possibility, and in another study, the authors found seropositivity against seasonal H3N2 virus was a significant risk factor for workers being seropositivity to swine H3N2 virus (Ma et al. 2015).

### Pathogenicity and transmission to humans

Although several human deaths have resulted from SIV infection (Tang et al. 2010; Short et al. 2015), the majority of SIV human infections are mild and indistinguishable from other seasonal influenza virus infections. Influenza H1N1pdm09 and H3N2 virus variants in the USA are the most recent swine-origin influenza viruses. Human mortality of influenza H1N1pdm09 was approximately 29 deaths per 100,000 infections, and among the 350 human cases of H3N2 variants, only one patient with unspecified concurrent diseases died (Tang et al. 2010; Short et al. 2015). Similar spill-over infection from pig to human were also reported in Europe. In 2016, a child in the Netherlands was infected by swine influenza A (H1N1) via contacting pigs (Fraaij et al. 2016). Another case was reported in Italy and the patient also had severe infection (Rovida et al. 2017). It worth noting that the two patients got severe syndromes, although all of his close contacts were not infected.

### Infection pathways: risk factors for human infection

The most common pathway for swine-to-human spread of SIV is exposure to live pigs. A study reported that exposure to pigs increased the chance of humans being infected with H3N2 SIV (OR = 3.05, 95% CI: 1.65–5.64) and working in large breeding herds also increased the likelihood of detecting anti-SIV antibodies in pig farm workers (OR = 3.98, 95% CI: 1.00–15.86) (Lopez-Robles et al. 2012). A study in the USA reported that there were spatio-temporal associations between the number of pig farms within counties and the timing of human flu cases, with peak number of cases during years when SIV was present, indicating transmission between pigs and humans (Bowman et al. 2014a; Lantos et al. 2016).

### Prevention of spillover of SIVs to humans

As the circulation of influenza A viruses among pigs and humans is very complicated in terms of the interaction of the two species in different ecosystems, it is difficult to recommend effective measures to prevent the transfer of infection from pigs to humans. Dorjee et al. (2016) used mathematical modelling to demonstrate that minimizing influenza transmissibility at pig-human interface through good personal hygiene, avoiding direct contacts with sick pigs, and targeted vaccination of swine workers with protective vaccine strains had significant beneficial effects on reducing spillover to humans. They also evaluated different strategies to minimize the duration and size of outbreaks if a spillover event happened, and suggested that early detection and effective quarantine in humans had the greatest impact on the control of influenza spread. Their findings support putting more emphasis on early detection of SIVs with pandemic potential in pigs, and hence the need for strengthening the monitoring of gene recombination among SIVs. Previous studies in south China showed that SIV and human/avian influenza viruses were circulating in local pig population simultaneously, and workers in the local pig industry adopted minimal self-protection measures while contacting pigs due to insufficient knowledge about SI (Li et al. 2019; Li et al. 2020b; Ding et al. 2021). The authors suggested risk-based surveillance and intervention for SI control in south China and targeting of the key counties that supply pigs to live pig markets would help control the transmission of strains to humans (Li et al. 2020c; Ding et al. 2021).

## Control measures for influenza in pigs

### Vaccination

Vaccination against SI may protect pigs from infection and is commonly used in sows because it is believed piglets are protected through maternal immunity to homologous influenza A virus strains (Chamba Pardo et al. 2019). Allerson et al. (2013a) demonstrated that vaccination of sows could significantly reduce SIV transmission among piglets; however, there are several challenges with SIV vaccination. Firstly, as homologous antibody against circulating strains is vital for the efficacy of vaccination in the field, it is critical to vaccinate with the current circulating strains. However, as different strains are commonly found in herds throughout the world, the failure of vaccination to induce protective immunity by not incorporating homologous local infecting strains in the vaccine cannot be ignored. Secondly, MDA may interfere with immunity against infection with homologous SIV strains in piglets, with one study showing that MDA in piglets could result in a prolonged shedding period of virus when piglets were subsequently infected with homologous SIV strains (Rose et al. 2013). Thirdly, vaccine-associated enhanced respiratory disease were observed in the field, which may further offset the benefit of using SIV vaccine (Mancera Gracia et al. 2020).

### Surveillance for swine influenza viruses

Surveillance programs for SIV have been developed and implemented in many countries. In the USA, the aims of SIV surveillance include protection of public health. However; detection, discovery and sharing of virus isolates to facilitate updates for vaccines, refine diagnostic assays, and determine the distribution of new influenza strains in pigs to inform further policy decisions are also advantages of this surveillance (Corzo et al. 2013a; Kaplan et al. 2015). In Europe, the European Surveillance Network for Influenza in Pigs (ESNIP, 2001–2012) was established to “increase the knowledge of the epidemiology and evolution of swine influenza virus in European pigs”. Most of the funds associated with this network have been directed towards undertaking research on antigenic and genetic characterization of field isolates of SIV (Detmer et al. 2013; Simon et al. 2014; Watson et al. 2015a).

For the purpose of preventing potential pandemic human influenza, it is valuable to monitor genetic drift, co-infection with different SIV subtypes on pig farms and emerging new reassortants of SIVs (Simon et al. 2014; Rajao et al. 2017). Thus, isolation, subtyping and gene sequencing of field strains are required. However, virological tests do not always detect field cases as nasal shedding of virus occurs for a limited period of time (Van Reeth et al. 2003; Hemmink et al. 2016), resulting in many affected pigs returning a virus-negative outcome. Furthermore, it is often difficult to culture SIVs and therefore subtype them when the viral load in samples is low. For example, Lopez-Robles et al. (2014) reported that even when clinical signs were present in 22 of 25 pigs that were positive for viral RNA, only isolates from 6 affected pigs were able to be subtyped by RT-PCR.

It is recommended that risk-based surveillance strategies are implemented to improve the efficiency of SIV surveillance. Risk-based surveillance is designed to detect pathogens or infections in the most likely places, herds or individuals, and thus can improve sensitivity of the surveillance system leading to more efficient use of resources and time (East et al. 2013). For example, more samples should be collected from pigs with suspicious clinical signs or a high risk of exposure to SIVs. Risk-based surveillance relies on knowledge about the diseases’ clinical signs, epidemiological characteristics, including the determinants for its spread and transmission (Stark et al. 2006; Oidtmann et al. 2013). Some studies have highlighted the advantages of risk-based surveillance for SI. For example, Li et al. (2020c) explored movement network of live pigs via live pig markets in south China, and the authors identified key areas that would have higher risk of having pathogens through the trading network. In addition, Ding et al. (2021) used existing SI surveillance data to develop machine learning models to predict the risks of human/avian influenza infection in pigs in different counties of south China.

Surveillance for influenza A viruses, including surveillance for SI, is in place in many countries (Kaden et al. 2008; Simon et al. 2014; Vincent et al. 2014; Kaplan et al. 2015). However, there is still room for improvement of SIV surveillance. Firstly, SI surveillance in key areas is insufficient. The surveillance capacity varies between countries, with many undeveloped countries having limited resources hindering their surveillance capacity. Secondly, the existing surveillance programs have not generated sufficient knowledge on the epidemiological features of SIV in different ecosystems. Thirdly, although passive surveillance is common in many countries, well-designed active surveillance is still rare. Passive surveillance may introduce bias in evaluating the presence and distribution of SIVs. Lastly, while more reassortants have been confirmed and compared by phylogenetic analysis, the relevant risk factors for continued circulation and persistent infection remain unclear (Trevennec et al. 2011; Vincent et al. 2014; Nelson et al. 2015b).

## Conclusions

Swine influenza can result in a significant economic loss for the pig industry and potentially lead to pandemic influenza in humans. Co-circulation of different SIV strains in pig farms can facilitate gene reassortment between strains, resulting in the production of new circulating strains in pigs and strains with pandemic potential. Certain husbandry and management practices and poor biosecurity on pig farms are risk factors for SIV infection in pig farms. Although reliable diagnostic tests for the disease are available, and many studies are focusing on monitoring the gene evolution of swine influenza viruses, disease control in pig farms is challenging. Close contact between pigs and workers in the pig industry offer opportunities for zoonotic transmission of swine influenza virus. To control the potential of swine-source flu pandemics developing in humans is a need for controlling swine influenza in pig farms.

## Data Availability

Not Applicable.

## Abbreviations

ADG: Average Daily Growth
cDNA: Complementary DNA
CI: Confidence Interval
ECEs: Embryonated Chicken Eggs
ELISA: Enzyme-Linked Immunosorbent Assay
FCE: Feed Conversion Efficiency
HA: Hemagglutinin
HI test: Hemagglutination Inhibition Test
HPAI: Highly Pathogenic Avian Influenza
MDA: Maternally Derived Antibody
MDCK: The Madin-Darby Canine Kidney
Mhp: *Mycoplasma hyopneumoniae*
NA: Neuraminidase
NP: Nucleoprotein
OR: Odds Ratio
RT-PCR: Reverse Transcription-Polymerase Chain Reaction Test
RT-qPCR: Quantitative Reverse Transcription PCR
SI: Swine Influenza
SIV: Swine Influenza Virus
SNA: Social Network Analysis
UK: The United Kingdom
USA: The United States Of America
VI: Virus Isolation
